# Large-Area Deposition of Hydrophobic Poly(hexafluorobutyl Acrylate) Thin Films on Wetting-Sensitive and Flexible Substrates via Plasma-Enhanced Chemical Vapor Deposition

**DOI:** 10.3390/polym17060791

**Published:** 2025-03-17

**Authors:** Kurtuluş Yılmaz, Mehmet Gürsoy, Mustafa Karaman

**Affiliations:** Chemical Engineering Department, Konya Technical University, Konya 42030, Türkiye; e208132002002@ktun.edu.tr (K.Y.); mkaraman@ktun.edu.tr (M.K.)

**Keywords:** PECVD, PHFBA, thin film, hydrophobic, plasma polymerization

## Abstract

In this study, hydrophobic poly(hexafluorobutyl acrylate) (PHFBA) thin films were successfully deposited over a large area of 25 × 50 cm using plasma-enhanced chemical vapor deposition (PECVD). Key parameters, including plasma power and the distance between the plasma antenna and the substrate, were optimized to achieve the highest deposition rate while ensuring uniformity and defect-free coatings. The optimal conditions were determined as 5 W plasma power and a 9 cm antenna–substrate distance, yielding a maximum deposition rate of 11.3 nm/min. PHFBA’s low fluorine content makes it a more environmentally and biologically friendly alternative compared to heavily fluorinated polymers, addressing concerns about toxicity and environmental impact. The coatings were applied to a flexible and wetting-sensitive paper towel substrate, which was successfully coated without any visible defects. The contact angle measurements confirmed the hydrophobic nature of the films, with a maximum water contact angle of 131.9° after the deposition of PHFBA. This study highlights the potential of PECVD as an efficient and scalable method for producing hydrophobic coatings, combining high-performance properties with improved environmental considerations. The results not only validate PECVD as a scalable and precise method for thin film fabrication but also open new possibilities for its use in applications requiring durable and functional surface modifications.

## 1. Introduction

The compatibility of hydrophobic polymers with various surfaces, such as paper, textiles, plastics, and metals, enable their use in numerous areas of daily life and industrial applications. Hydrophobic polymer coatings are utilized in the fabrication of microfluidic devices, filtration systems, biomedical materials, and textiles [1,2,3,4]. Fluoropolymers are employed as hydrophobic polymers due to their lower surface energies, low dielectric constant values, and chemical inertness compared to hydrocarbon polymers [5].

Long-chain fluorochemicals, such as per- and polyfluoroalkyl substances (PFASs), possess a wide range of applications due to their chemical and thermal stability. However, these chemicals pose potential threats to both ecosystems and human health because of their resistance to biological degradation [6,7]. It has been demonstrated that as the length of the fluoroalkyl chains increases, the toxicity and bioaccumulation potential of fluorochemicals also rise [8]. Considering these risks associated with traditionally used fluoropolymers with long perfluorinated chains, short-chain fluorochemicals have emerged as an alternative that minimizes adverse environmental impacts. Consequently, there has been a growing interest in fluoropolymers with low fluorine content in recent years [9,10].

Poly(hexafluorobutyl acrylate) (PHFBA) represents a promising alternative to traditional fluorochemicals due to its low fluorine content and excellent water-repellent properties [11,12]. Additionally, it has been found that PHFBA thin films are non-toxic to human epithelial cells, even after a four-day period, with high cell viability rates. This study demonstrated that cell viability is comparable to that of control cells, indicating that PHFBA thin films can be considered non-toxic [13]. Fluoropolymer thin films can be fabricated using various methods [14,15,16,17,18]. Plasma-enhanced chemical vapor deposition (PECVD) has emerged as a versatile technique for producing thin films. Compared to traditional coating methods, PECVD offers several advantages [19]. For instance, dip-coating methods may struggle with inconsistent coverage. Similarly, spray-coating methods often result in material waste and uneven layers. PECVD overcomes these limitations by providing nanoscale precise layer control and the ability to create uniform coatings, including on complex and porous substrates [20,21]. It also results in reduced material waste. Additionally, as a gas-phase method, PECVD inherently eliminates the use of solvents, making the process environmentally friendly. The PECVD method operates at low temperatures, allowing for the coating of heat- and moisture-sensitive materials, such as textiles and paper, without damaging their surfaces [22,23]. CVD processes can be easily scaled to achieve high efficiency and large-scale production. For example, polymeric thin film coatings have been applied on a large scale by integrating roll-to-roll and reel-to-reel equipment into CVD processes or by using large vacuum chambers [24,25]. The main objective of this study is to demonstrate the industrial-scale production potential of PHFBA thin films produced using a PECVD system. In this scope, hydrophobic PHFBA thin films were applied to large-area (50 cm × 25 cm) substrates. Key PECVD parameters, including plasma power and the distance between the plasma antenna and the substrate, were optimized to achieve the highest deposition rate while ensuring uniformity and defect-free coatings. In addition, the large-area contact angle and thickness uniformity of the PHFBA thin film were also investigated. Therefore, for the first time in the literature, PECVD process parameters were optimized for the large-area deposition of fluorinated polymers having hydrophobic functionality. The as-deposited large-area films can be applicable in a wide range of areas, including lab-on-a-chip, biosensing, tissue engineering, membrane filtration, and so on.

## 2. Materials and Methods

### 2.1. Materials

The HFBA (95%) monomer was purchased from Sigma-Aldrich. The monomer was introduced directly into the vacuum chamber without any purification or modification. The substrates used in the study included silicon wafers (University Wafer, <100>, p-type), paper towel (made of 100% cellulose, ARO, İzmir, Türkiye) and glass slide (Marienfeld, Lauda-Königshofen, Germany).

### 2.2. Method

**Figure 1:**
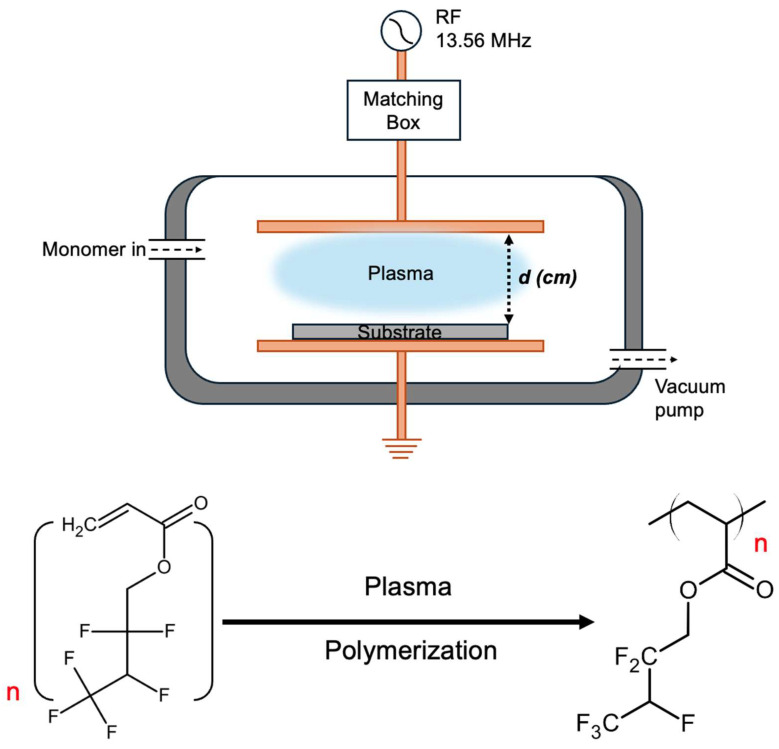
The schematic representation of the PECVD system, along with the reaction scheme of PHFBA polymerization (the red n denotes the number of repeating monomer units).

The deposition of PHFBA thin films was performed in a custom-built cylindrical vacuum chamber with a diameter of 75 cm and a height of 12 cm. A schematic representation of the experimental setup is provided in Figure 1. A rotary vacuum pump (2XZ-15C, EVP, Shanghai, China) was employed to establish the vacuum conditions necessary for polymerization. The HFBA liquid monomer was evaporated at room temperature within a stainless-steel jar and introduced into the vacuum chamber at a controlled flow rate of 1 sccm through a needle valve (Swagelok, Solon, OH, USA) during the polymerization process. To ensure thermal stability, the reactor wall temperature was maintained at 40 °C using heater cables connected to a variac unit (adjustable autotransformer). A vacuum gauge (925 Micro Pirani, MKS instruments, Andover, MA, USA) was utilized to monitor the pressure inside the vacuum chamber, with all depositions conducted at a pressure of 100 mTorr. The activation energy required for polymerization was supplied by a plasma discharge generated within the vacuum chamber. For this purpose, parallel copper plates (55 cm × 25 cm), connected to a 13.56 MHz RF power source, were positioned inside the vacuum chamber to facilitate plasma generation. Two independent parameters were investigated in this study. The first parameter was the applied plasma power, which was adjusted to six different levels between 1 W and 20 W with an electrode-substrate separation distance of 4.5 cm. The second parameter was the distance between electrode and substrate, which was varied across six different distances ranging from 1.5 cm to 9 cm under an applied plasma power of 5 W. The effects of these parameters on the deposition process were systematically analyzed.

### 2.3. Characterizations

The chemical structure of the as-deposited PHFBA films on the glass substrate was analyzed using Fourier Transform Infrared Spectroscopy (FTIR, Thermo Fisher Scientific, Nicolet iS20, Loughborough, UK) with an ATR accessory. FTIR measurements were conducted in the wavenumber range of 500–2750 cm⁻^1^ at a resolution of 4 cm⁻^1^ with 32 scans. Additionally, X-ray Photoelectron Spectroscopy (XPS, Thermo Fisher Scientific K-Alpha XPS, Loughborough, UK) was utilized to further characterize the chemical composition of PHFBA films deposited on a silicon wafer. High-resolution XPS spectra were acquired to analyze the binding energies of chemical structures, using high-resolution modes with a pass energy of 30 eV and a step size of 0.1 eV. The optical transmittance of uncoated and PHFBA-coated glass was measured using a UV/Vis spectrophotometer (Shimadzu UV-1800, Shimadzu Inc., Kyoto, Japan) over the wavelength range of 400–800 nm. The water contact angle measurements were performed on a PHFBA-coated paper towel using a contact angle goniometer (Kruss Easy Drop, Hamburg, Germany) with 4 μL water droplets. The surface morphology of both uncoated and PHFBA-coated paper towels was examined via Scanning Electron Microscopy (SEM, Zeiss EVO LS-10, Oberkochen, Germany). The final thickness of the as-deposited thin film on 1 × 1 cm^2^ silicon wafer was measured ex situ using a thin film reflectometer (Avantes, Avaspec-ULS2048L, Beijing, China). The deposition rates were found by dividing the final film thickness to the deposition time for each run. The surface roughness of the PHFBA-coated silicon wafer was examined using Atomic Force Microscopy (AFM, TT-2 AFM Workshop, Moscow, Russia) in a semi-contact mode. Measurements were performed over a 5 × 5 µm^2^ area, and alongside surface imaging, the roughness values were calculated. Adhesion testing of PHFBA films deposited on 1 × 1 cm^2^ silicon wafer surfaces was performed five times using a 3M Scotch 600 tape (MN, USA) test. First, a diagonal scratch pattern was created on the coated surface using a flat-tipped scalpel. The adhesive tape was then firmly applied to the scratched area and quickly removed at 90° angle. The surface images before and after tape application were analyzed using an optical microscope (iSCOPE, Euromex, Arnhem, The Netherlands).

## 3. Results and Discussion

### 3.1. Deposition Rates

**Figure 2:**
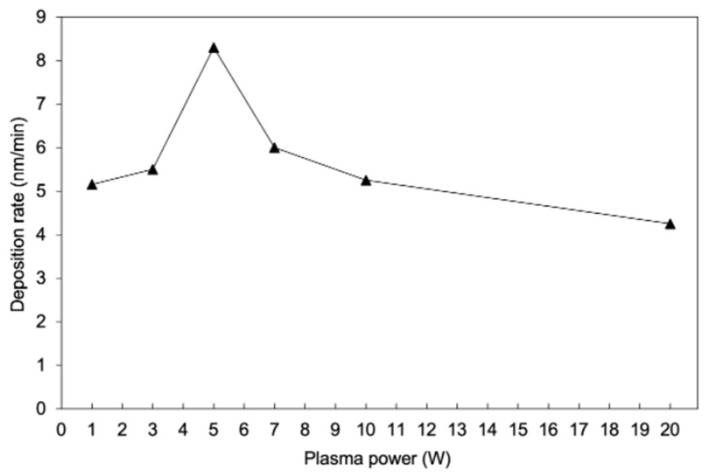
Deposition rates of PHFBA deposited at different plasma powers (4.5 cm distance between the electrode and substrate).

Figure 2 shows the influence of plasma power on the PHFBA deposition rate. The deposition of PHFBA films using plasma polymerization could be achieved at very low applied plasma powers, starting at even 1 W. With an increase in the applied plasma power, the deposition rates increased from 1 W to 5 W. However, a further increase in the applied power up to 20 W caused a decrease in the deposition rates.

**Figure 3:**
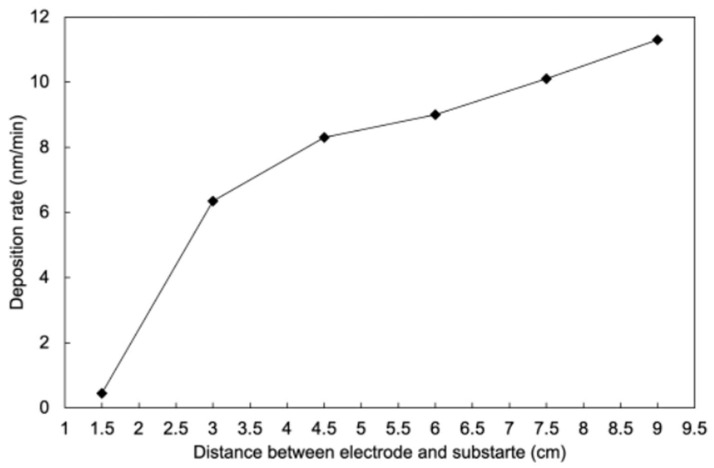
Deposition rates of PHFBA deposited at different distances between the electrode and substrate (5 W plasma power).

In plasma polymerization, two competing mechanisms, namely deposition and etching, may cause the changes in the overall deposition rates [26,27]. Going from 1 W to 5 W increases the deposition rate through the generation of more active gaseous species that take part in the thin film formation process. However, with a further increase in the plasma power, the extensive generation of active species, especially highly energetic fluorinated moieties, may initiate the onset of the etching process, which would decrease the overall deposition rates. Additionally, the bombardment of the substrate surface with energetic ions facilitates the ablation process, which may be another plausible reason behind the decreased deposition rates at higher plasma powers [28]. Figure 3 shows the influence of the distance between electrode and substrate on the PHFBA deposition rate. These experiments were performed at an applied plasma power of 5 W, at which the highest deposition rate was observed. With an increase in the separation distance, the deposition rates increased continuously. The low deposition rates at a low substrate–electrode separation can be associated with the presence of a sheath at the electrode, i.e., a region of low or no glow near the electrode. With an increase in the distance, the substrates can be exposed to the glow region without the plasma sheath, which may contribute to the improved deposition rates [29]. Of course, a further increase in the separation distance may decrease the deposition rates after reaching a certain threshold distance, which we could not measure due to the size limit of our reactor set-up.

### 3.2. Film Structure

**Figure 4:**
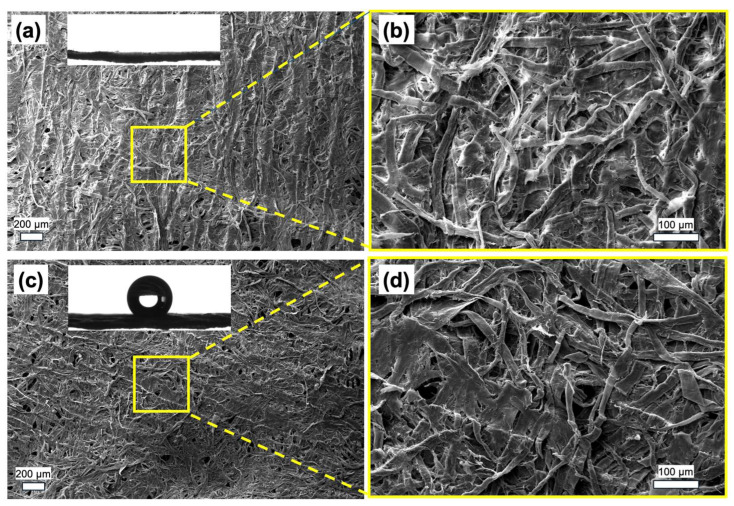
The SEM images of (**a**,**b**) uncoated and (**c**,**d**) PHFBA-coated paper towels, along with their corresponding contact angle measurements (5 W plasma power and 9 cm distance between the electrode and substrate).

The structural analyses of the as-deposited films were carried out using the films deposited under a plasma power of 5 W and electrode separation of 9 cm, at which the highest deposition rate was observed. In order to obtain the same film thicknesses for structural analyses, deposition durations were kept constant at 20 min. The PHFBA thin film coating was successfully applied to wetting-sensitive paper towel surfaces. No defects were detected by the naked eye after coating. To examine the changes in surface morphology in more detail, SEM analysis was performed before and after coating, and the results are presented in Figure 4a,b and Figure 4c,d, respectively. SEM images were acquired at magnifications of 250× and 1000×. The SEM images confirm that the surfaces are homogeneous and uniformly coated. In addition, the contact angle results were incorporated into the corresponding SEM images. Before coating, the paper towel completely absorbed water, whereas after PHFBA thin film deposition, it exhibited hydrophobic properties with a measured contact angle of 131.9°.

**Figure 5:**
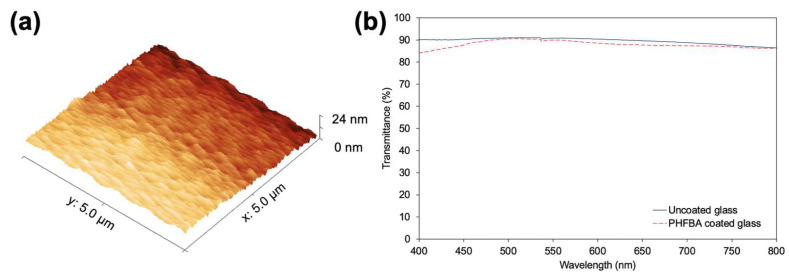
(**a**) AFM images of PHFBA-coated silicon wafer; (**b**) optical transmittances of PHFBA-coated and uncoated glasses (5 W plasma power and 9 cm distance between the electrode and substrate).

The AFM image in Figure 5a shows the surface morphology of the PHFBA thin film coated on the silicon wafer surface. As a result of the analysis, the root mean square (Rq) and average (Ra) roughness values of the surface were calculated as 4.21 nm and 3.60 nm, respectively. These values indicate that the film formation was homogenous and the surface was quite smooth. In addition, the low roughness values obtained are consistent with the polymeric thin films produced by CVD methods in the literature [30,31].

The optical transmittance in the visible region of the PHFBA thin film-coated glass was compared with uncoated glass (Figure 5b). The results reveal that the PHFBA thin film has high optical transmittance. The use of bulk polymers in applications requiring optical transmittance is often challenging. However, the high optical transmittance of the PHFBA thin film suggests that it may be a suitable alternative for such applications. Furthermore, the low surface roughness observed in the AFM results indicates that there are no large irregularities or microscopic scattering centers on the surface of the film. This may have allowed light to pass through the surface in an orderly manner, thus reducing optical losses and contributing to the achievement of high transmittance [32,33]. It is clearly seen from Figure 5b that both uncoated and coated glass substrates have very similar transmittance values in a wide range of wavelength values. Indeed, the as-deposited PHFBA thin film may act as an antireflective surface since its refractive index value (~1.4) is between that of air and glass. It is known that the decrease in the overall reflectance indicates an increase in the total transmittance. Therefore, at some wavelength values, such as 500 nm, the transmittance values can show no change or even be better for the coated glass. The oscillations of the transmittance values in the visible range may be caused by the optical interference phenomenon. The initial decrease in transmittance may be due to the heterogeneous thickness of the film and different absorption coefficients for different wavelengths [34].

**Figure 6:**
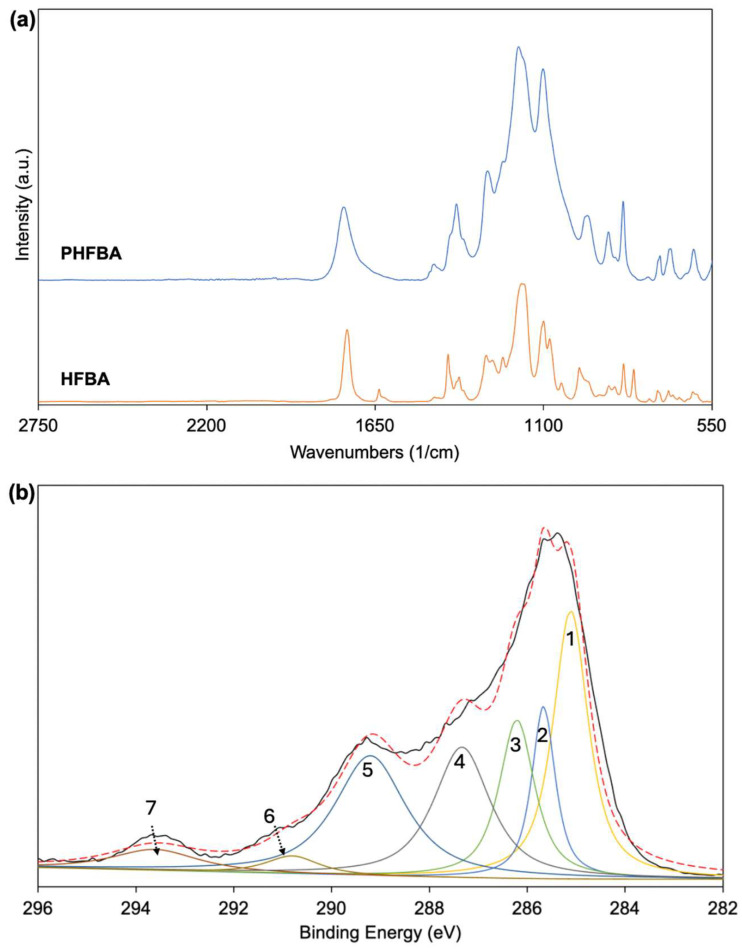
(**a**) FTIR spectra of HFBA and PHFBA; (**b**) high-resolution XPS C1s spectrum of PHFBA. The corresponding chemical bonds and binding energies of the peaks numbered 1–7 in (**b**) are provided in Table 1. The solid black line represents the experimental data, while the red dashed line shows the overall fitted curve obtained from peak deconvolution. (5 W plasma power and 9 cm distance between the electrode and substrate).

Figure 6a presents the comparative FTIR spectra of the HFBA monomer and the PHFBA thin film deposited on the glass substrate. Both spectra were normalized to the intensity of the C=O stretching peak at 1753 cm⁻^1^. The peaks observed at 2980 and 2889 cm⁻^1^ correspond to asymmetric and symmetric CH_2_ stretching vibrations, respectively. The peak at 1457 cm⁻^1^ is attributed to CH_2_ bending vibrations, while the peaks at 1384, 1283, 1181, and 1101 cm⁻^1^ correspond to symmetric CF_3_ stretching, CF_2_ vibrations, CF stretching, and CF_2_ stretching vibrations, respectively, confirming the presence of fluorine-containing functional groups in the structure. Additionally, the peaks at 967, 888, and 840 cm⁻^1^ are associated with the bending vibrations of CF_2_ and CF_3_ groups. The peaks in the range of 768–530 cm⁻^1^ correspond to the CF deformation vibrations of fluorinated groups. Notably, the absence of the characteristic C=C stretching peak at 1637 cm⁻^1^, which is present in the HFBA monomer spectrum, in the PHFBA thin film confirms that polymerization occurs through the reaction of unsaturated C=C double bonds [11,35,36].

As seen in Figure 6b, the high-resolution C(1s) spectrum of PHFBA thin films reveals seven distinct bonding states: C*F_3_ (293.6 eV), C*F_2_ (290.8 eV), C*=O (289.2 eV), CH_2_-CF_2_-C*HF (287.3 eV), O-C*H_2_ (286.2 eV), C*H-CO (285.6 eV), and C-C*H_2_-C (285.1 eV) [37,38]. The observed binding energies in the C1s spectrum, along with their assigned functional groups and corresponding theoretical values, are presented in Table 1.

Some deviations were observed in the FTIR and XPS results of PHFBA. However, as reported in previous studies on PECVD-based polymerization, such variations are commonly encountered in plasma-assisted polymer deposition processes. It is well known that monomers exposed to high plasma power can undergo excessive ion, electron, and UV bombardment, leading to disordered bonding configurations or the partial loss of functional groups [39,40,41]. This effect arises due to the energetic nature of the polymerization process, which can induce structural modifications in the deposited thin films. Despite these deviations, the FTIR spectrum obtained in this study suggests that the essential functional groups of the monomer were largely preserved in the PHFBA thin film. Similarly, the binding energies of the bonds observed in the XPS spectrum were found to be in close agreement with their theoretical values, further supporting the successful deposition of the PHFBA thin film.

### 3.3. Large-Scale Depostion

**Figure 7:**
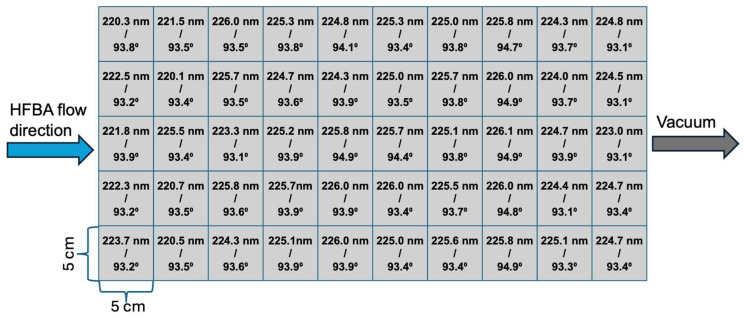
Large-area thickness and contact angle results of PHFBA-coated silicon wafer (5 W plasma power and 9 cm distance between the electrode and substrate).

**Figure 8:**
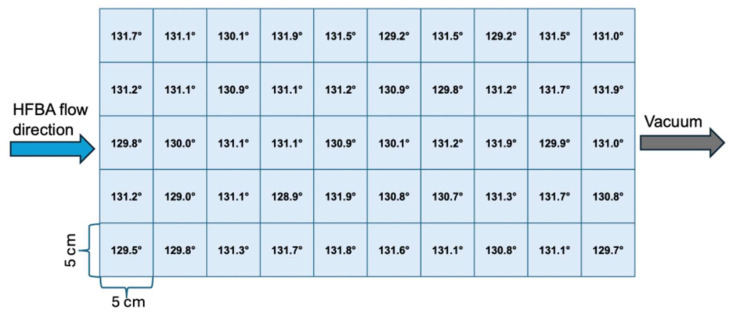
Large-area contact angles results of the PHFBA-coated paper towel (5 W plasma power and 9 cm distance between the electrode and substrate).

In this study, we aimed to demonstrate the uniform deposition of PHFBA thin films over a large area. For this purpose, the 50 cm × 25 cm reactor floor was virtually divided into 50 distinct zones, each with an area of 5 cm^2^. Silicon wafers and a paper towel were placed as substrates in each of these zones. The distance between the electrode and the substrates was maintained at 9 cm, and the PHFBA thin film was deposited at a 5 W plasma power for 20 min. Film thickness measurements were obtained from silicon wafer surfaces, while contact angle measurements were taken from both the paper towel and silicon wafer surfaces. The measured film thicknesses and contact angle values are schematically presented in Figure 7. The water contact angle on the paper towel substrate surfaces are presented in Figure 8.

The film thicknesses ranged between 220.1 nm and 226.1 nm, with an average thickness of 224.49 nm and a standard deviation of 1.65 nm. Contact angle measurements were conducted on both silicon wafer and paper towel surfaces. On the silicon wafer, the average contact angle was 93.73° with a standard deviation of 0.51°, with recorded minimum and maximum values of 93.1° and 94.9°, respectively. On the paper towel, the average contact angle was 130.85°, with a standard deviation of 0.83°, and values ranging between 128.9° and 131.9°. The low standard deviation values in both measurements indicate high uniformity. The results reveal that the coating thickness exhibits only a 0.73% variation from the mean value, while the contact angle varies by just 0.54% on the silicon wafer surfaces and 0.63% on paper towel surfaces. These findings confirm that the PHFBA thin film is highly uniform in terms of both thickness and wettability characteristics.

**Figure 9:**
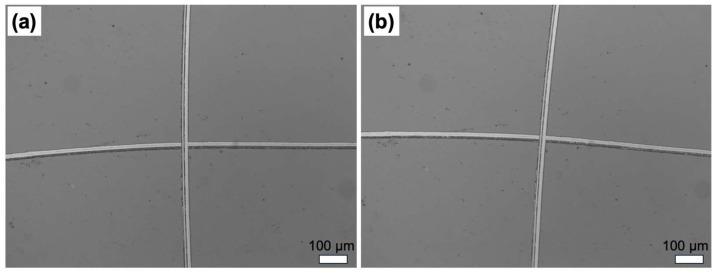
Ten-times-magnified optical microscope images of the surfaces (**a**) before and (**b**) after tape application (5 W plasma power and 9 cm distance between the electrode and substrate).

The mechanical durability of thin films is crucial for their long-term performance, especially in applications where they are exposed to mechanical stress. The adhesion test results (Figure 9a,b) demonstrate that the PHFBA thin films deposited via PECVD exhibit strong mechanical stability on silicon wafer surfaces. The absence of any observable deformation after the adhesion test indicates high durability, which can be attributed to the cross-linked structure of the film formed during the plasma polymerization process [42,43]. These findings suggest that the PECVD-coated films possess excellent mechanical robustness, making them suitable for applications requiring long-term durability under mechanical stress.

## 4. Conclusions

This study demonstrates the successful large-area deposition of hydrophobic PHFBA thin films using PECVD, highlighting its effectiveness in achieving uniform, defect-free coatings. The ability to coat wetting-sensitive and flexible substrates, such as paper towels, without compromising their integrity underscores the versatility of this approach. Unlike heavily fluorinated polymers, PHFBA provides a more environmentally responsible alternative, balancing hydrophobic performance with reduced ecological impact. The negligible variation in both the thickness (0.73%) and contact angle (0.63%) further confirms the high precision and repeatability of the PECVD process. The successful coating of porous and flexible substrates expands the applicability of PHFBA thin films in fields such as next-generation packaging, wearable technology, biomedical devices, and filtration systems.

## Figures and Tables

**Table 1:** Observed and theoretical binding energies of C1s peaks in PHFBA thin films.

C1s	Origin	Binding Energy (eV) Theoretical	Binding Energy (eV) Experimental
1	C-C*H_2_-C	285.0	285.1
2	C*H-CO	285.7	285.6
3	O-C*H_2_	286.7	286.2
4	CH_2_-CF_2_-C*HF	286.8	287.3
5	C*=O	289.2	289.2
6	C*F_2_	291.2	290.8
7	C*F_3_	293.3	293.6

## Data Availability

The original contributions presented in this study are included in the article. Further inquiries can be directed to the corresponding author.
